# Association Between Gut Microbiota and *Helicobacter pylori*-Related Gastric Lesions in a High-Risk Population of Gastric Cancer

**DOI:** 10.3389/fcimb.2018.00202

**Published:** 2018-06-19

**Authors:** Juan-Juan Gao, Yang Zhang, Markus Gerhard, Raquel Mejias-Luque, Lian Zhang, Michael Vieth, Jun-Ling Ma, Monther Bajbouj, Stepan Suchanek, Wei-Dong Liu, Kurt Ulm, Michael Quante, Zhe-Xuan Li, Tong Zhou, Roland Schmid, Meinhard Classen, Wen-Qing Li, Wei-Cheng You, Kai-Feng Pan

**Affiliations:** ^1^Key Laboratory of Carcinogenesis and Translational Research (Ministry of Education), Department of Cancer Epidemiology, Peking University Cancer Hospital and Institute, Beijing, China; ^2^Institute of Medical Microbiology, Immunology and Hygiene, Technische Universität München, Munich, Germany; ^3^German Center for Infection Research, Partner Site Munich, Munich, Germany; ^4^Institute of Pathology, Klinikum Bayreuth, Bayreuth, Germany; ^5^II. Medizinische Klinik, Klinikum Rechts der Isar, Technische Universität München, Munich, Germany; ^6^Department of Internal Medicine, 1st Faculty of Medicine, Charles University, Military University Hospital, Prague, Czechia; ^7^Linqu Public Health Bureau, Linqu, Shandong, China; ^8^International Digestive Cancer Alliance, Munich, Germany

**Keywords:** *Helicobacter pylori*, gastric lesions, gut microbiota, microbial diversity, 16S ribosomal RNA gene sequencing

## Abstract

Eradication of *Helicobacter pylori* has been found to be effective for gastric cancer prevention, but uncertainties remain about the possible adverse consequences such as the potential microbial dysbiosis. In our study, we investigated the association between gut microbiota and *H. pylori*-related gastric lesions in 47 subjects by deep sequencing of microbial 16S ribosomal RNA (rRNA) gene in fecal samples. The dominant phyla in fecal samples were *Bacteroidetes, Firmicutes*, and *Proteobacteria* with average relative abundances of 54.77, 31.37 and 12.91%, respectively. Microbial diversity analysis showed that observed species and Shannon index were increased in subjects with past or current *H. pylori* infection compared with negative subjects. As for the differential bacteria, the average relative abundance of *Bacteroidetes* was found to significantly decrease from *H. pylori* negative (66.16%) to past infection group (33.01%, *p* = 0.007), as well as from normal (76.49%) to gastritis (56.04%) and metaplasia subjects (46.83%, *p* = 0.027). For *Firmicutes* and *Proteobacteria*, the average relative abundances showed elevated trends in the past *H. pylori* infection group (47.11, 20.53%) compared to negative group (23.44, 9.05%, *p* = 0.068 and 0.246, respectively), and similar increased trends were also found from normal (18.23, 5.05%) to gastritis (35.31, 7.23%, *p* = 0.016 and 0.294, respectively) or metaplasia subjects (32.33, 20.07%, both *p* < 0.05). These findings suggest that the alterations of fecal microbiota, especially the dominant phyla of *Bacteroidetes, Firmicutes* and *Proteobacteria*, may be involved in the process of *H. pylori*-related gastric lesion progression and provide hints for future evaluation of microbial changes after *H. pylori* eradication.

## Introduction

Gastric cancer (GC) is a major health problem in China, accounting for over 40% of the new GC cases annually worldwide (Torre et al., 2015). In the multistep gastric carcinogenesis process, *Helicobacter pylori* plays a crucial role by inducing inflammation and degradation of the gastric epithelium from early stage (Correa, 1992). The factors affecting the outcome of *H. pylori* infection are diverse and include bacterial virulence, host genetic factors and host microbiota (Lofgren et al., 2011).

The human gastrointestinal microbiota is a complex and dynamic ecosystem regarded as a metabolically active “organ.” Thus, cosstalk between microbes and immune cells regulates human gastrointestinal homeostasis (Mortha et al., 2014). The balance of commensal microbiota in the gastrointestinal tract may play important roles for regulation of host mucosal immune response, energy metabolism, elimination of pathogens and cancer development (Garrett, 2015; Rooks and Garrett, 2016). Many previous studies suggested that gastric microbiota can be affected by *H. pylori* infection (Maldonado-Contreras et al., 2011; Jo et al., 2016; Schulz et al., 2018). Dysbiosis of gastric microbiota and some specific bacteria were found to be associated with GC or precancerous lesions (Eun et al., 2014; Coker et al., 2018; Ferreira et al., 2018). In addition, a distinct shift of microbiota in the distal intestinal tract was reported following hypochlorhydria and hypergastrinemia by *H. pylori* infection in Mongolian gerbils (Heimesaat et al., 2014). However, little is known about the association between intestinal microbiota and *H. pylori* infection or GC and precancerous lesions in human beings. Since it is well appreciated that microbiota composition shape immune responses at a local and systemic level, and also that GC development is influenced by inflammatory signaling, it is tempting to speculate that *H. pylori* associated alterations of the gut microbiota may in turn influence GC development.

*H. pylori* eradication was found to be effective in preventing precancerous gastric lesions and GC in previous intervention trials (Wong et al., 2004; You et al., 2006). However, uncertainties remain about the possible adverse consequences of anti-*H. pylori* treatment. Several studies suggested that adverse health effects, including obesity (Ley et al., 2006), asthma (Couzin-Frankel, 2010), or other immunological conditions (Sekirov et al., 2010) may be associated with the perturbation of gut microbiome after *H. pylori* eradication. Thus, a deep understanding of the association between microbial dysbiosis and *H. pylori*-related precancerous gastric lesions is urgently needed for optimizing the efficacy of GC prevention strategies.

In the present study, we analyzed the gut microbiota of subjects presenting gastric lesions associated with or independent of *H. pylori* infection by deep sequencing of microbial 16S ribosomal RNA (rRNA) gene in feces. Our results provide baseline associations between gut microbiota and *H. pylori*-related gastric lesions, which will be important for further evaluation of eradication treatment and gastric carcinogenesis.

## Materials and methods

### Study subjects and sample collection

In November 2014, an endoscopy study was conducted in Linqu County, China. A total of 218 asymptomatic individuals (aged 40–69 years) from two villages of Yishan Township were selected for ^13^C-urea breath test (^13^C-UBT) and standardized upper endoscopy examination. The exclusion criteria were as follows: contraindication for biopsy sampling, risk of conscious sedation due to severe comorbidities, and inability to provide an informed consent due to psychiatric illness. In the final week of the endoscopic examinations, participants were invited to provide stool samples. A total of 47 participants agreed and provided samples, which were collected and frozen immediately at −80°C until DNA extraction. Besides, each participant completed a physical examination and a questionnaire on age, gender, cigarette smoking, alcohol consumption, and antibiotics treatment history under the supervision of a well-trained interviewer before the endoscopic examination. This study was approved by the Institutional Review Boards of Peking University Cancer Hospital and Institute and written informed consent was obtained from all of the participants.

### Upper endoscopic examination and histopathology

Upper endoscopic examinations were conducted by five international experienced gastroenterologists using video endoscopes (Olympus). The gastric mucosa was examined, and five or more biopsies were obtained from antrum, corpus, angulus, cardia, esophagus and any suspicious lesions. All gastric mucosa specimens were reviewed blindly by two pathologists (1 Chinese and 1 German) according to the Updated Sydney System (Dixon et al., 1996). Each biopsy was scored using a visual analog scale (0 = absent, 1 = mild, 2 = moderate, 3 = severe) for *H. pylori* infection, gastritis, activity of inflammation and intestinal metaplasia (Dixon et al., 1996). Each subject was assigned a global diagnosis for each variable based on the most severe diagnosis reported from all the biopsies.

### Determination of *H. pylori* infection status

*H. pylori* infection status was determined according to ^13^C-UBT and histological diagnosis. ^13^C-UBT was performed as reported previously (Pan et al., 2016). Subjects with concentration of ^13^CO_2_ that exceeded the baseline >3.8 parts per 1000 (>0.38%) after 30 min were regarded as positive. Current *H. pylori* infection was defined when bacteria were histologically detected in any one of the biopsies or it was positively indicated by ^13^C-UBT. Non-current infection subjects were defined when both bacterial and ^13^C-UBT detection indicated negative results.

From the non-current infection subjects, past infection subjects were identified by record on previous *H. pylori* eradication therapy or histological diagnosis of non-active gastritis with basal lymphoid aggregates and reactive changes of the surface epithelium in antrum and corpus, indicating a prior *H. pylori* infection. Non-current infection subjects without eradication treatment history and specific histological diagnosis were considered negative *H. pylori* infection subjects.

### Nucleic acid extraction from stool samples

Fecal DNA was extracted using the Qiagen Fast DNA Stool Mini Kit (Qiagen, MD, USA) according to the protocol provided by the manufacturer. Briefly, 180–220 mg sample was re-suspended in InhibitEX Buffer to adsorb DNA-damaging substances and PCR inhibitors. After thoroughly homogenizing, the suspension was heated to 95°C for 5 min to maximize the lysis of bacteria. The quantity and quality of total DNA were verified by NanoDrop-2000 spectrophotometer (Thermo Scientific) and 1% standard agarose gel electrophoresis.

### 16S rRNA gene sequencing

For analysis of microbial composition, the hypervariable region V4 of microbial 16S rRNA gene was amplified using Phusion® High-Fidelity PCR Master Mix (New England Biolabs) and the universal primers (515F, 5′-GTGCCAGCMGCCGCGGTAA-3′; 806R, 5′-GGACTACHVGGGTWTCTAAT-3′) (Baker et al., 2003; Huws et al., 2007). Thirty cycles were performed. Then PCR products were analyzed by 2% agarose gel electrophoresis, pooled in equidensity ratios, and purified with GeneJET Gel Extraction Kit (Thermo Scientific). Sequencing libraries were generated using NEB Next® Ultra® DNA Library Prep Kit for Illumina (NEB, USA) following manufacturer's recommendations. Library quality was assessed using a Qubit@ 2.0 Fluorometer (Thermo Scientific) and an Agilent Bioanalyzer 2100 system. The library was sequenced on an Illumina MiSeq platform (Novogene Bioinformatics Technology Co., Ltd. Beijing, China) to produce 250-bp paired-end reads.

### Data and statistical analysis

Paired-end reads were merged by FLASH (V1.2.7) (Magoc and Salzberg, 2011). The raw sequences were filtered to obtain high-quality sequences according to QIIME (Version 1.7.0) (Bokulich et al., 2013). UCHIME algorithm (Edgar et al., 2011) was used to detect and remove chimeras. Sequences with ≥97% similarity were assigned to the same operational taxonomic units (OTUs) by UPARSE software package (UPARSE, v7.0.1001) (Edgar, 2013). A representative sequence for each OTU was picked and annotated using the Mothur method based on the SSUrRNA database of SILVA (Quast et al., 2013) (setting a threshold of 0.8–1) to obtain classification levels (phylum, class, order, family, genus and species). The abundance information of OTUs was normalized with a standard sequence number corresponding to the sample with the fewest sequences, based on which subsequent analyses were performed.

The microbial alpha diversity of fecal samples was illustrated by observed species and Shannon index, which were calculated using QIIME (Version 1.7.0). Rarefaction curves were displayed with R software (Version 2.15.3). Comparisons of microbial communities were visualized by heat-map and Principal Co-ordinates Analysis (PCoA) based on weighted unifrac distances between clinical parameters and gut microbiota, and the significance of differences was evaluated by the analysis of similarity (ANOSIM) depending on the Bray-Curtis dissimilarity distance matrices. Taxa with relative abundance more than 0.1% in at least one group (without special emphasis) were included in identification of differential taxa. Welch's *t*-test was utilized to identify differences in the average relative abundances of individual taxonomy between two groups (Parks et al., 2014).

Continuous variables, such as age, body mass index (BMI) and fasting plasma glucose, were reported as mean ± standard deviation, while observed species and Shannon index were reported as median (interquartile range, IQR). Categorical variables (gender, smoking, drinking, antibiotics treatment history and gastric lesions) were described as frequencies and proportions. Comparisons of continuous parameters were performed by Mann-Whitney test between two groups or Kruskal-Wallis test among three groups. Fisher's exact test was conducted for comparisons between categorical variables. Statistical analysis was carried out using SPSS 13.0 (SPSS, USA), and *p* < 0.05 were considered statistically significant.

## Results

From 47 subjects who provided fecal samples, 24 subjects were identified as having current *H. pylori* infection and 23 subjects as non-current infection, of which 15 were *H. pylori* negative and 8 showed signs of past infection (Table 1). The frequency of metaplasia was marginally higher in the current *H. pylori* infection and in the past infection groups than in the negative group (62.50, 50.00, and 20.00%, *p* = 0.065). No significant differences were found among the three groups in age, gender, BMI, smoking, drinking, fasting plasma glucose and antibiotics use (all *p* > 0.05).

**Table 1:** Characteristics of the participants included in this study.

**Characteristics**	**Total, *n* = 47 *n* (%)**	***H. pylori*** **status**			
		**Negative, *n* = 15**	**Past infection, *n* = 8**	**Current infection, *n* = 24**	***p***
		***n* (%)**	***n* (%)**	***n* (%)**	
Age, years (Mean ± SD)	52.68 ± 7.10	53.53 ± 8.79	55.50 ± 7.71	51.21 ± 5.48	0.382^a^
Gender					0.423^b^
Male	17 (36.17)	4 (26.67)	2 (25.00)	11 (45.83)	
Female	30 (63.83)	11 (73.33)	6 (75.00)	13 (54.17)	
BMI, kg/m^2^ (Mean ± SD)	22.42 ± 3.03	21.59 ± 2.05	23.04 ± 3.75	22.73 ± 3.30	0.538^a^
FPG, mmol/L (Mean ± SD)	5.61 ± 0.64	5.78 ± 0.68	5.40 ± 0.42	5.58 ± 0.68	0.393^a^
Smoking					0.999^b^
No	40 (85.11)	13 (86.67)	7 (87.50)	20 (83.33)	
Yes	7 (14.89)	2 (13.33)	1 (12.50)	4 (16.67)	
Drinking					0.724^b^
No	36 (76.60)	12 (80.00)	7 (87.50)	17 (70.83)	
Yes	11 (23.40)	3 (20.00)	1 (12.50)	7 (29.17)	
Antibiotics use within 1 month					0.870^b^
No	31 (65.96)	9 (60.00)	5 (62.50)	17 (70.83)	
Yes	13 (27.66)	5 (33.33)	2 (25.00)	6 (25.00)	
Missing	3 (6.38)	1 (6.67)	1 (12.50)	1 (4.17)	
Gastric lesions					0.065^b^
Normal	7 (14.89)	4 (26.67)	0 (0)	3 (12.50)	
Gastritis	18 (38.30)	8 (53.33)	4 (50.00)	6 (25.00)	
Metaplasia	22 (46.81)	3 (20.00)	4 (50.00)	15 (62.50)	

a *Kruskal Wallis Test*.

b *Fisher's Exact Test*.

*BMI, body mass index; FPG, fasting plasma glucose; H. pylori, Helicobacter pylori; SD, standard deviation*.

### Fecal microbiota composition

A total of 2,827,158 paired-end reads were generated from 47 fecal samples including 2,557,652 quality-filtered reads with an average of 54,418 ± 5,665 (standard deviation) reads per sample. After aligning to the SILVA database and removing non-bacterial sequences, 2,518,760 (98.48%) of the quality-filtered reads were finally retained for subsequent analysis with an average of 53,591 ± 5,625 reads per sample, which generated 817 OTUs at 97% similarity level. The rarefaction curves showed a reasonable sequencing depth (Figure S1).

**Figure 1:**
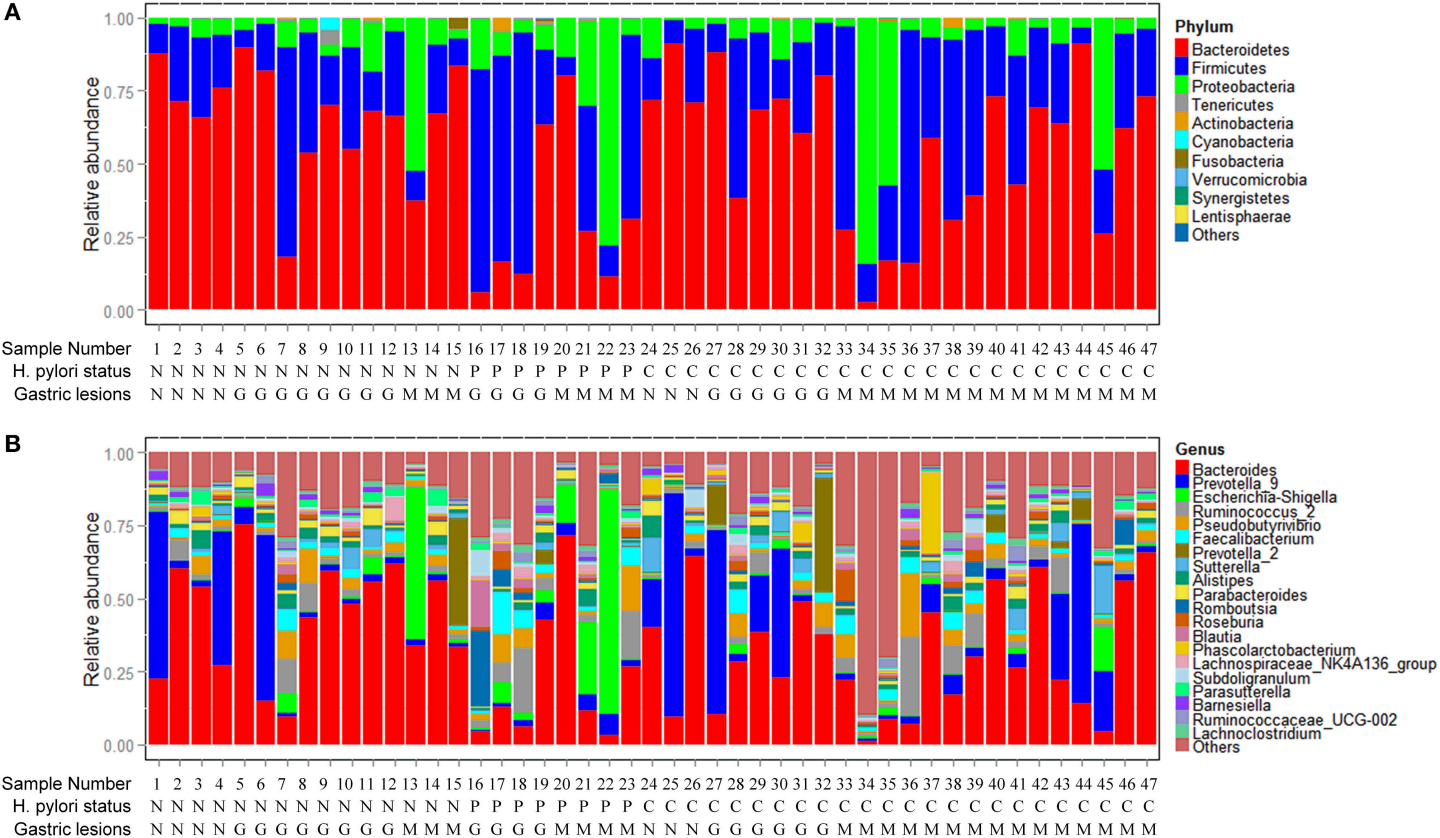
Composition of fecal microbiota. **(A)** Relative abundance distribution of major phyla (top 10) in 47 fecal samples. Phyla were sorted by decreasing order of average relative abundance. *H. pylori* status and gastric lesions observed for each subject are listed below. **(B)** Relative abundance distribution of major genera (top 20) in 47 fecal samples. Genera were sorted by decreasing order of average relative abundance as in **(A)**. *H. pylori* status: N, *H. pylori* negative; P, past *H. pylori* infection; C, current *H. pylori* infection. Gastric lesions: N, normal; G, gastritis; M, metaplasia.

Phyla and genera of fecal microbiota detected in the 47 subjects are summarized in Figure 1. The most abundant phyla were *Bacteroidetes, Firmicutes*, and *Proteobacteria* with average relative abundances of 54.77%, 31.37% and 12.91%, respectively. The three dominant phyla accounted for 99.05% of all fecal bacteria (Figure 1A). At the genus level, fecal microbiota was dominated by *Bacteroides, Prevotella*_9, *Escherichia-Shigella* and *Ruminococcus*_2 with average relative abundances of 33.53, 12.55, 5.07, and 4.34%, respectively (Figure 1B). Only one sequence of *Archaea* was found in two of the fecal samples with relative abundances <0.10% (Table S1).

### Association between *H. pylori* infection and fecal microbiota

To explore the association between *H. pylori* infection and fecal microbiota, we firstly compared the microbial alpha diversity, community structure, and differential taxa between current and non-current *H. pylori* infection groups. For alpha diversity analysis, we used observed species to evaluate microbial richness and Shannon index to evaluate evenness. No statistical differences were found in medians (IQR) of observed species [316.00 (285.75–351.50) vs. 324.00 (289.00–351.00), *p* = 0.831] and Shannon index [4.83 (3.73–5.36) vs. 4.79 (3.99–5.44), *p* = 0.983] between non-current *H. pylori* infection and current infection group (Figures S2A,B). Likewise, no significant difference in fecal bacterial community structure was found between the current and non-current *H. pylori* infection groups (*R* = 0.007, *p* = 0.300, Table S3; Figure S2C).

When we compared the differential taxa between the two groups, no significant differences were found in major phyla. At the genus level, average relative abundances were found significantly decreased for *Acidovorax* and *Rhodococcus* (*p* = 0.016 and 0.017, respectively), and increased for *Gemella* and *Erysipelotrichaceae_UCG_004* (*p* = 0.002 and 0.020, respectively) in the current infection group when compared to the non-current infection group (Figure S2D). However, the average relative abundances of these differential genera were only <0.1% of the fecal microbiota.

**Figure 2:**
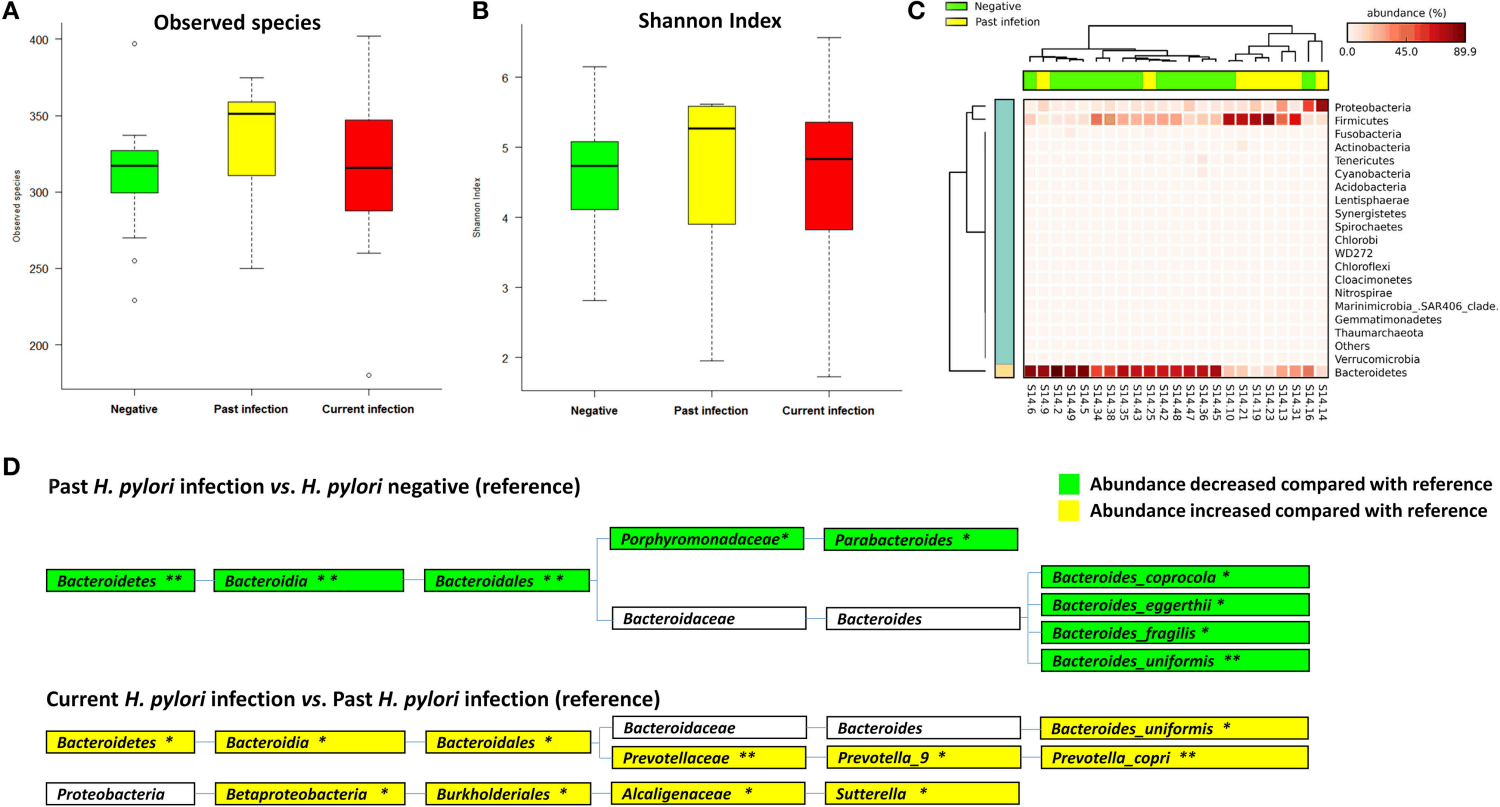
Associations between *Helicobacter pylori* infection and fecal microbiota. **(A)** Boxplot of observed species in different *H. pylori* status groups. The boxes indicate interquartile ranges (IQRs) and the median (blank line).Whiskers extend to the most extreme points within 1.5-fold IQR. Outliers are plotted individually (°). **(B)** Shannon index in different *H. pylori* status groups. **(C)** Heat-map of relative abundance distributions of the phyla in past *H. pylori* infection (*n* = 8) and negative groups (*n* = 15). **(D)** Cladogram of differently distributed taxa between groups. The differential taxa are illustrated between past *H. pylori* infection and negative groups, or current *H. pylori* infection and past infection groups as ^*^p < 0.05 and ^**^p < 0.01, respectively.

Non-current *H. pylori* infection subjects were divided into negative (*n* = 15) and past infection (*n* = 8) groups according to antibiotics treatment history and histological assessment to further compare the fecal microbiota among the different groups. Observed species [median (IQR) 317.00 (289.00–328.00)] and Shannon index [4.74 (3.99–5.27)] were increased from negative, to past infection [351.00 (296.00–362.00), 5.27 (3.46–5.59)] or current infection group [316.00 (285.75–351.50), 4.83 (3.73–5.36)], but the differences were not statistically significant (*p* = 0.317 for observed species and 0.696 for Shannon index) (Table S2, Figures 2A,B).

A significant difference in fecal microbial community structure was found by ANOSIM between past *H. pylori* infection and negative groups (*R* = 0.316, *p* = 0.004, Table S3, Figure S3A), while no statistical difference was found between the current *H. pylori* infection and negative groups (*R* = −0.038, *p* = 0.763, Table S3, Figure S3A). The heat-map based on relative abundances of all phyla also revealed that past *H. pylori* infection subjects were distinct from subjects of the negative group (Figure 2C).

We further studied differential bacteria among the three different *H. pylori* infection groups (Figure 2D). The average relative abundance of *Bacteroidetes* (including the genera of *Parabacteroides* and some species belonging to *Bacteroides*) was significantly decreased from 66.16% in the *H. pylori* negative group to 31.01% in the past infection group, *p* = 0.007. The average relative abundance of *Bacteroidetes* was also found increased from 31.01% in the past *H. pylori* infection group to 55.57% in the current infection group, *p* = 0.043. Especially *Bacteroides uniformis* species showed significantly decreased relative abundance in the past *H. pylori* infection group (1.50%), when compared to the current infection (3.74%, *p* = 0.022) and the negative groups (5.37%, *p* = 0.002), respectively.

### Association between gastric lesions and fecal microbiota

**Figure 3:**
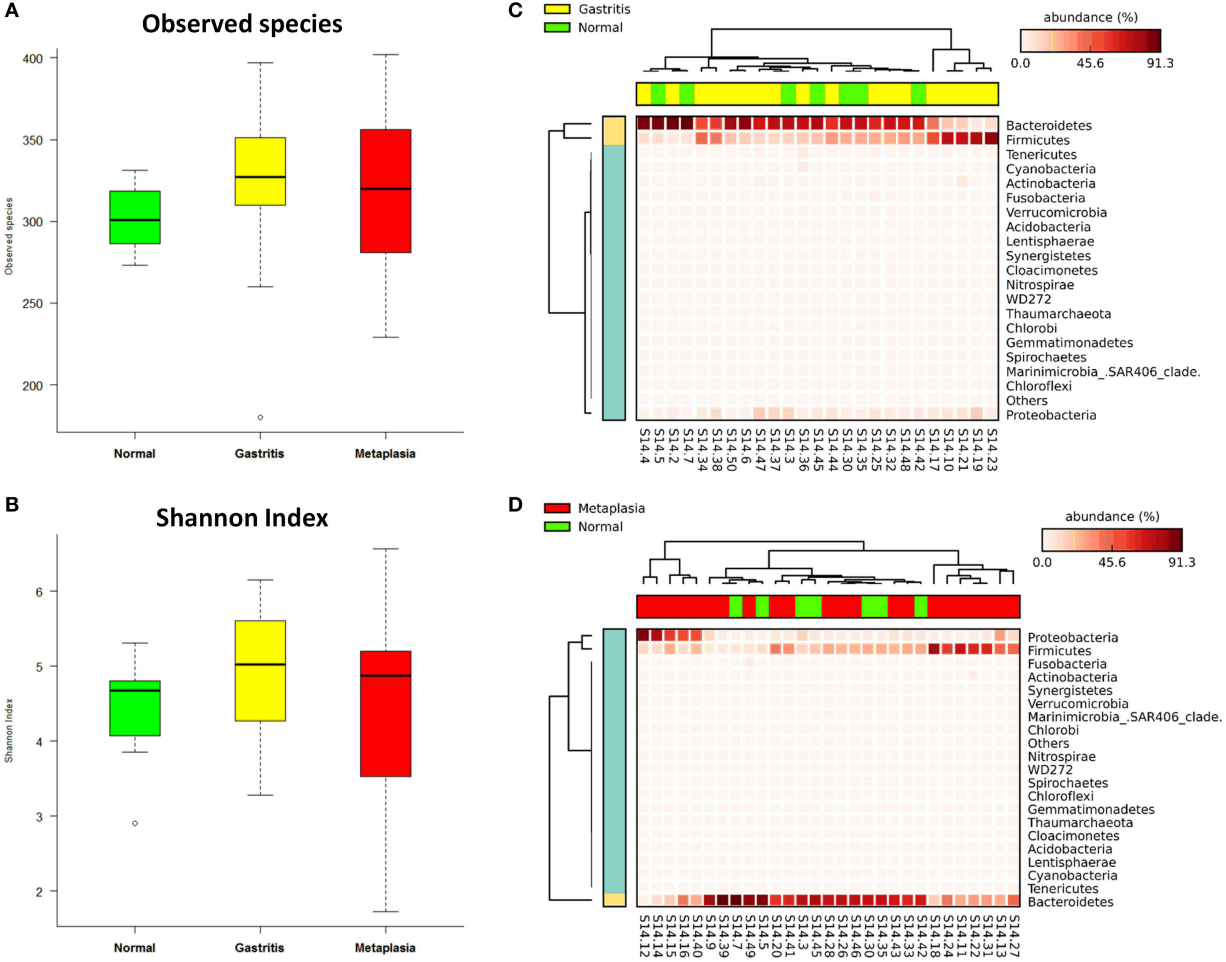
Associations of gastric lesions with fecal microbiota. **(A)** Boxplot of observed species in different gastric lesion groups. **(B)** Boxplot of Shannon index in different gastric lesion groups. **(C)** Heat-map demonstrating the relative abundance of different phyla in gastritis (*n* = 18) and normal (*n* = 7) samples. **(D)** Heat-map showing the relative abundance distributions of different phyla in metaplasia (*n* = 22) and normal samples.

The global gastric pathological diagnosis of the 47 subjects included in the study ranged from normal to gastritis and metaplasia. This provided the opportunity to investigate the relationship between gastric lesions and fecal microbiota. Microbial alpha diversity analysis revealed no significant differences in observed species and Shannon index medians among the normal [301.00 (284.00–320.00), 4.68 (3.85–4.84)], gastritis [327.00 (309.75–351.50), 5.03 (4.26–5.61)] and metaplasia groups [320.00 (281.00–358.00), 4.87 (3.42–5.26), both *p* > 0.05] (Table S2). When compared to the normal group as reference, observed species and Shannon index were increased in gastritis and metaplasia groups, but the differences were not statistically significant (*p* = 0.397 in gastritis, *p* = 0.759 in metaplasia for observed species, and *p* = 0.258, *p* = 0.983 for Shannon index) (Figures 3A,B).

No significant difference in fecal microbial community structure was found among the different gastric lesions groups by ANOSIM (*R* = −0.029, *p* = 0.704, Table S3, Figure S3B), while the heat-map based on the relative abundance of all phyla still showed differences between normal subjects and cases with gastritis or metaplasia (Figures 3C,D).

**Figure 4:**
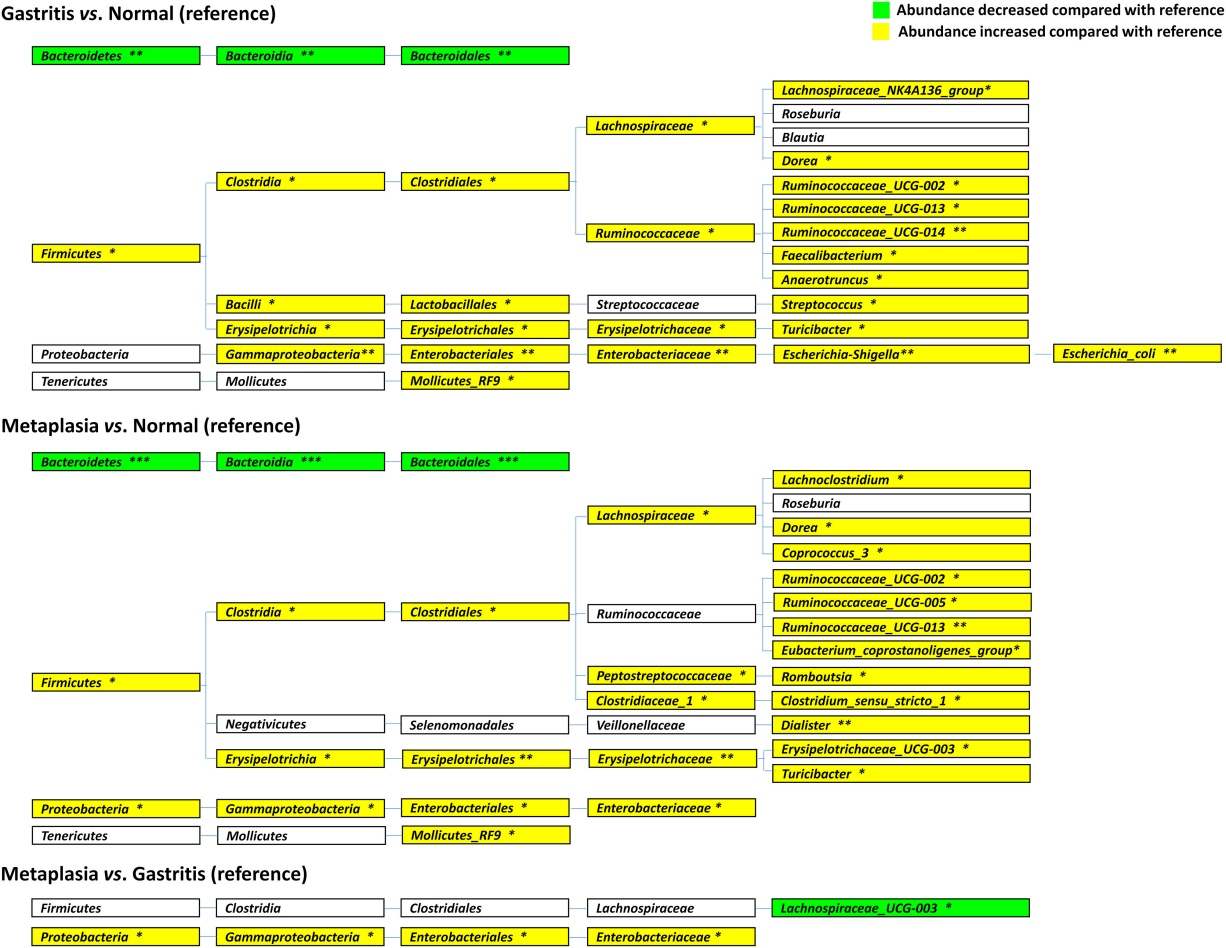
Cladogram of differentially distributed taxa among different gastric lesion groups. The differential taxa are illustrated between gastritis and normal, metaplasia and normal, or metaplasia and gastritis groups as ^*^p < 0.05, ^**^p < 0.01, and ^***^p < 0.001, respectively.

The differential bacteria observed in the different gastric lesions groups are shown in Figure 4. Compared to normal subjects, the differential phyla of fecal microbiota in gastritis cases were *Bacteroidetes*, with decreased average relative abundance from 76.49% (normal) to 56.04% (gastritis), *p* = 0.009, and *Firmicutes* with increased average relative abundance from 18.23 to 35.31%, *p* = 0.016. When we compared metaplasia cases with normal subjects, similar significant differences in average relative abundances were also found for *Bacteroidetes* (metaplasia: 46.83% vs. normal: 76.49%, *p* < 0.001) and *Firmicutes* (metaplasia: 32.33% vs. normal: 18.23%, *p* = 0.016). In addition, *Proteobacteria* showed significantly increased average relative abundance from normal (5.05%) to metaplasia subjects (20.07%, *p* = 0.016) and from gastritis (7.23%) to metaplasia (20.07%, *p* = 0.034). Finally, the average relative abundance of *Enterobacteriaceae*, a family of *Proteobacteria*, was significantly increased with the severity of the gastric lesions analyzed (normal: 0.60%, gastritis: 2.95%, metaplasia: 16.14%, *p* = 0.033).

### Association between activity of gastritis and fecal microbiota

Since neutrophil activity is a very sensitive indicator for current *H. pylori*-related gastritis, we further analyzed the association between activity of gastritis and fecal microbiota. Of the 18 subjects diagnosed with gastritis, 12 presented non-active gastritis and 6 showed active gastritis. Analyses of observed species and Shannon index showed that the microbial richness and evenness in fecal samples were slightly increased in non-active gastritis subjects [332.50 (312.25–352.50), 5.03 (4.72–5.61)] compared to normal subjects [301.00 (284.00–320.00), 4.68 (3.85–4.84)], although *p* values were 0.098 and 0.184, respectively (Table S2, Figures S4A,B). No significant differences were found between subjects presenting active gastritis [316.00 (240.00–346.25), 4.81 (3.55–5.46)] and normal subjects [301.00 (284.00–320.00), 4.68 (3.85–4.84), *p* = 0.803, and 0.739, respectively].

No significant differences among normal, non-active and active gastritis groups (*R* = 0.063, *p* = 0.194) were detected when comparing the fecal microbiota community structure by ANOSIM (Table S3) and heat-map analysis (Figures S4C,D). In contrast, for differential taxa selection, the average relative abundance of *Ruminococcaceae_NK4A214_group* was found decreased in active gastritis (0.06%) compared to non-active gastritis subjects (0.16%, *p* = 0.030) (Figure S4E).

### Identification of relevant fecal microbial alterations in *H. pylori* infection and gastric lesions

**Figure 5:**
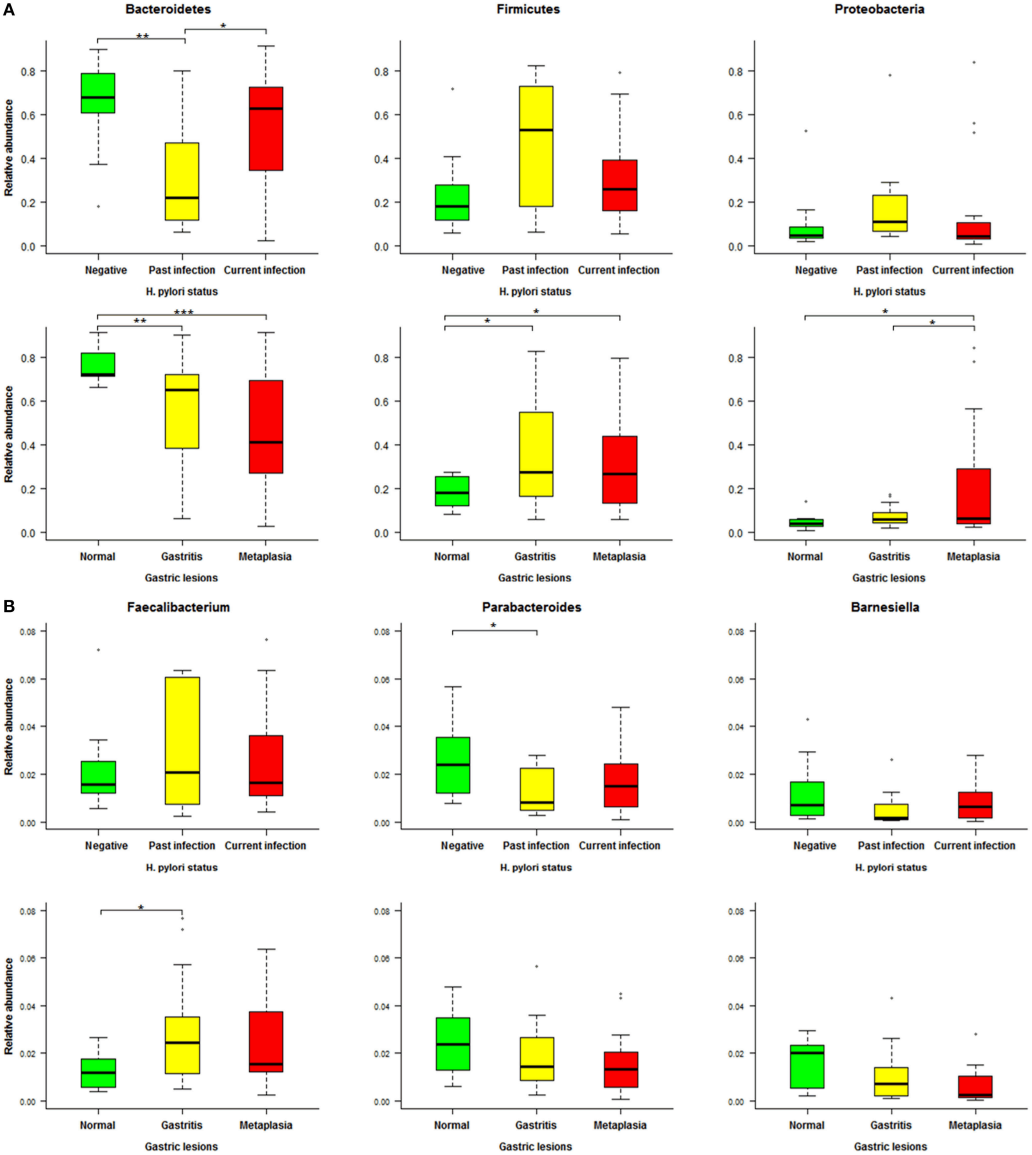
Relative abundance distributions of specific phyla and genera in different *Helicobacter pylori* infection status and gastric lesion groups. **(A)** Boxplot of the main phyla in different *H. pylori* infection status and gastric lesion groups. **(B)** Boxplot of specific genera in different *H. pylori* infection status and gastric lesion groups. Significant differences between groups are indicated as ^***^p < 0.001, ^**^p < 0.01, and ^*^p < 0.05, respectively.

To further identify associations between fecal microbiota and *H. pylori*-related gastric lesions, we finally analyzed relevant microbial alterations in relation to *H. pylori* infection and progression of gastric lesions. In the three dominant phyla (*Bacteroidetes, Firmicutes*, and *Proteobacteria*) of fecal microbiota (Figure 5A), the average relative abundance of *Bacteroidetes* decreased from normal (76.49%) to gastritis (56.04%) and metaplasia (46.83%, *p* = 0.027), as well as from *H. pylori* negative (66.16%) to past infection (33.01%, *p* = 0.007). For *Firmicutes* and *Proteobacteria*, the average relative abundances were increased from normal (18.23, 5.05%) to gastritis (35.31, 7.23%, *p* = 0.016 and 0.294) or metaplasia (32.33, 20.07%, both *p* < 0.05), respectively. Similarly, the average relative abundances of these two phyla were found elevated in past *H. pylori* infection (47.11, 20.53%) compared to the negative group (23.44, 9.05%), although the *p* values (0.068 and 0.246) showed no significant differences.

At the genus level, a total of 19 genera with average relative abundances of more than 0.01% were analyzed in relation to *H. pylori* infection status and gastric lesions. Three genera (*Parabacteroides, Barnesiella*, and *Faecalibacterium*) were found to have similar alterations in *H. pylori* infection and gastric lesion groups, although most of the *p* values were higher than 0.05 among groups with different *H. pylori* infection status or gastric lesions (Figure 5B). The average relative abundances of *Parabacteroides* and *Barnesiella* (belonging to the phylum of *Bacteroidetes*) decreased with the severity of gastric lesions (normal: 2.48 and 1.56%, gastritis: 1.97 and 1.01%, metaplasia: 1.56 and 0.61%, respectively), as well as *H. pylori* infection status (negative: 2.44 and 1.15%, past infection: 1.27 and 0.58%, respectively). In addition, *Faecalibacterium* (belonging to the phylum of *Firmicutes*) showed a trend to higher average relative abundance both in the gastritis (3.45%) and the past *H. pylori* infection groups (4.04%) compared to their reference (normal: 1.27%, negative: 2.07%), respectively.

## Discussion

Deep understanding of the association between gut microbiota and *H. pylori*-related precancerous gastric lesions is very important for evaluation of overall benefits and adverse effects of eradication treatment and for the optimization of GC prevention strategies. In the present study, we profiled fecal microbiota in subjects with different *H. pylori* infection status. Diversity analysis showed slightly increased microbial richness and evenness in subjects presenting signs of past *H. pylori* infection or gastritis compared to controls. In addition, the relative abundances of the dominant phyla *Bacteroidetes, Firmicutes*, and *Proteobacteria* in fecal microbiota were similarly altered in subjects presenting with *H. pylori* infection and gastric precancerous lesions. These observations suggest that fecal microbiota may be associated with the progression of *H. pylori*-related gastric lesions.

The human gut is inhabited by a huge number of microorganisms, the composition of which was considered critical for maintenance of gastrointestinal homeostasis. The gut microbiota is quite stable within an individual, while it varies extremely inter-individually. Many factors were reported to influence the gut microbiota including antibiotics treatment and diet (Francino, 2015; Bajaj et al., 2018). When the microbial balance or “symbiosis” turns to “dysbiosis” by the influence of various factors, the normal cohabitants of the gut may transform into “pathobionts” and trigger carcinogenesis by inducing chronic inflammation and the activation of immune mechanisms (Tözün and Vardareli, 2016). Several gastric and intestinal microbes have been recently shown as procarcinogens in GC and colorectal cancer (Jo et al., 2016; Wang et al., 2016; Flemer et al., 2017; Coker et al., 2018; Ferreira et al., 2018), or probiotics enhancing immunotherapy response of cancer patients (Gopalakrishnan et al., 2018), while little has been reported about microbiota composition in precancerous lesions. In the present study we describe the composition of fecal microbiota in a high-risk population showing different precancerous gastric lesions and *H. pylori* infection status. We found that the dominant phyla present in feces were *Bacteroidetes, Firmicutes*, and *Proteobacteria*, accounting for 99.05% of fecal microbiota, which is consistent with previous reports (Tözün and Vardareli, 2016). Although *Helicobacter* species was reported to dominate gastric microbiota in actively infected subjects, we only found few *Helicobacter* specific sequences in fecal samples of *H. pylori* positive subjects, which may be due to the different environment of the intestinal tract, in which *H. pylori* does not survive.

*H. pylori* colonizes the gastric epithelium and may influence GC progression through the interaction with gastric microbiota, which was supported by the observation of enhanced gastric carcinogenesis in *H. pylori*-infected germfree INS-GAS mice colonized only with three commensal bacteria (ASF356 *Clostridium*, ASF361 *Lactobacillus murinus* and ASF519 *Bacterioides*) (Lertpiriyapong et al., 2014). Many human studies showed that gastric microbiota can be altered by *H. pylori* infection (Schulz et al., 2018), with relatively lower diversity and differential abundances of *Proteobacteria, Firmicutes*, and *Actinobacteria* (Bik et al., 2006; Maldonado-Contreras et al., 2011; Li et al., 2017). Although studies on associations between *H. pylori* infection and intestinal microbiota were sparse, some bacteria were reported to be related with *H. pylori*, including *Bacteroides, Prevotella* spp., *Clostridium histolyticum*, and *Lactobacilli* in animal models and human studies (Bühling et al., 2001; Myllyluoma et al., 2007; Heimesaat et al., 2014). In our population-based study, only a minority of genera (*Acidovorax, Rhodococcus, Gemella*, and *Erysipelotrichaceae_UCG-004*) in feces was found associated with current *H. pylori* infection. No significant differences in fecal microbial diversity and structure were found between subjects showing current or non-current *H. pylori* infection groups. These observations are in line with the results of a previous small sample size study showing no remarkable alterations of the fecal microbiota composition and structure after *H. pylori* eradication (Yap et al., 2016). However, our present pilot baseline study still needs validation in a subsequent larger sample size prospective trial.

In the non-current infection subjects identified by ^13^C-UBT and histologic assessment, we further diagnosed past *H. pylori* infection from negative cases by evaluating eradication treatment history or the presence of non-active gastritis with basal lymphoid aggregates and reactive changes on the surface epithelium, which may implicate prior *H. pylori* infection. When we compared the three *H. pylori* infection groups, the distributions of possible confounding factors (including age, gender, smoking, drinking, fasting plasma glucose, BMI and recent antibiotics use) showed no significant differences. Especially, the recent antibiotics use (within 1 month) was not significantly different among negative (33%), past infection (25%), and current infection groups (25%). Consistent with a mouse model study (Lofgren et al., 2011), we found a trend to relatively increased microbial diversity and decreased relative abundance of *Bacteroidetes* in past *H. pylori* infection group compared to negative and current infection cases, respectively. The similar decrease of *Bacteroidetes* 12 months after *H. pylori* eradication in a Malaysian study (Yap et al., 2016) further suggests that long-term observation should be considered to establish associations between *H. pylori* infection and changes in intestinal microbiota.

So far, there are few studies about the associations between gastric microbiota and precancerous gastric lesions. A metagenomic analysis found that gastric microbial diversity was gradually decreased from gastritis to intestinal metaplasia and GC (Aviles-Jimenez et al., 2014), while other studies showed that in *H. pylori* positive gastric mucosa microbial diversity was higher in GC than in gastritis and intestinal metaplasia (Eun et al., 2014; Wang et al., 2016). Recently, some specific bacteria including *Peptostreptococcus stomatis, Slackia exigua, Parvimonas micra, Streptococcus anginosus*, and *Dialister pneumosintes* were found to be associated with gastric carcinogenesis (Coker et al., 2018). However, no studies were yet conducted to investigate possible alterations in intestinal microbiota in relation to gastric lesions. Our study shows a slight increased trend of microbial diversity in fecal samples of subjects presenting gastritis and metaplasia when compared to individuals showing no pathological changes in the gastric mucosa. This may be related to the fact that more *H. pylori* affected subjects (current and past infection) were included in the gastric lesion groups than in the normal mucosa group. The significantly different taxa observed between different lesion groups suggest a possible role of intestinal microbiota in the progression of precancerous gastric lesions. Altered microbiota observed include the dominant phyla of *Bacteroidetes* and *Firmicutes*, as well as relevant gut bacteria such as *Enterobacteriaceae* and *Faecalibacterium* previously reported to be related to inflammatory diseases such as primary biliary cholangitis (Tang et al., 2018). Of note, the frequencies of previous antibiotics treatment in our study were relatively higher in gastritis (77.8%) and metaplasia groups (68.2%) than in normal (28.6%), implicating that our results still require further validation with a larger sample size.

The mechanisms by which gut microbiota contribute to carcinogenesis are still not clear. Dysbiosis of microbiota can affect the host immune system, inducing inflammation and thereby the development of cancer due to the tight interplay between bacteria and epithelial cells. Innate bacteria-sensing receptors such as Toll-like receptors (TLRs) and NOD-like receptors (Rakoff-Nahoum and Medzhitov, 2009) may bridge this interplay, which eventually can promote carcinogenesis in a chronic process. These mechanisms may also be involved in the associations between fecal microbiota and *H. pylori* infection and gastric lesions observed in our population. The activity of gastritis is well known for its close relationship with *H. pylori* infection. This close relationship is further confirmed by the similar alterations that we observed in the fecal microbiota from non-active gastritis and past infection subjects. In addition, the same alteration tendencies detected for major phyla or genera, such as decreased abundance of *Bacteroidetes* and increased abundances of *Firmicutes* or *Proteobacteria*, with the severity of gastric lesion and *H. pylori* infection status (especially past infection status), further suggest that alterations in intestinal microbiota may be involved in the progression of *H. pylori*-related precancerous gastric lesions and carcinogenesis.

A major strength of our study lies in the detailed information on *H. pylori* infection status and the availability of tissue samples with a broad spectrum of gastric lesions from an ongoing intervention trial for *H. pylori* eradication. Exploration of the associations between fecal microbiota and *H. pylori* infection and gastric lesions builds a solid basis for our future study on the changes in microbiota after *H. pylori* eradication. A limitation of our study is that we only had fecal samples from 47 subjects after endoscopy examination. Therefore, the small sample size limited the feasibility to adjust possible confounding factors or correct multiple comparisons for the identification of differential taxa among groups with different *H. pylori* infection status or gastric lesions. However, possible factors influencing the intestinal microbiota were acquired in detail at baseline, including BMI, fasting plasma glucose and antibiotics use, which were equally distributed among different groups. Confirmation with a larger sample size including participants of the intervention trial as well as subjects showing precancerous lesions or GC is still needed. In addition, the composition of gastric microbiota and its association with *H. pylori*-induced gastric carcinogenesis will be further studied.

In conclusion, our study profiled the composition of fecal microbiota and assessed its association with *H. pylori* infection and gastric lesion status. The differential bacteria identified, including *Bacteroidetes, Firmicutes* and *Proteobacteria*, showed similar alterations associated with *H. pylori* infection and severity of the gastric lesions. Our results provide novel insights important for future studies on *H. pylori*-related carcinogenesis and alterations in the gut microbiota after *H. pylori* eradication.

## Data availability statement

The raw data of this manuscript will be made available by the authors, without undue reservation, to any qualified researcher.

## Author contributions

K-FP and W-CY contributed design of the study. MB and SS conducted upper endoscopy examination. LZ, J-LM, W-DL, and Z-XL contributed subject's recruitment and samples' collection. MV, KU, MQ, RS, and MC completed histological diagnosis. YZ, J-JG, and TZ carried out experiments. J-JG analyzed experiments' results. YZ and J-JG wrote the draft of the manuscript. MG, RM-L, W-QL and K-FP revised the manuscript. All authors read and approved the submitted version.

### Conflict of interest statement

The authors declare that the research was conducted in the absence of any commercial or financial relationships that could be construed as a potential conflict of interest.

## Abbreviations

^13^C-UBT: ^13^C-urea breath test
ANOSIM: analysis of similarity
BMI: body mass index
GC: gastric cancer
*H. pylori*: *Helicobacter pylori*
IQR: interquartile range
OTUs: operational taxonomic units
rRNA: ribosomal RNA
SD: standard deviation
vs.: versus.
