# Supplementary material for: Association Between Gut Microbiota and Helicobacter pylori-Related Gastric Lesions in a High-Risk Population of Gastric Cancer
Source: Front Cell Infect Microbiol. 2018 Jun 19;8:202. doi: 10.3389/fcimb.2018.00202 (PMC6018392; doi:10.3389/fcimb.2018.00202)
Supplement: Supplementary file 1 [file Data_Sheet_1.docx]

Supplementary Material

Association between gut microbiota and *Helicobacter pylori*-related gastric lesions in a high-risk population of gastric cancer

**Juan-juan Gao^1†^, Yang Zhang^1†^, Markus Gerhard^2,3^, Raquel Mejias-Luque^2,3^, Lian Zhang^1^, Michael Vieth^4^, Jun-Ling Ma^1^, Monther Bajbouj^5^, Stepan Suchanek^6^, Wei-Dong Liu^7^, Kurt Ulm^2^, Michael Quante^5^, Zhe-Xuan Li^1^, Tong Zhou****^1^, Roland Schmid^5^, Meinhard Classen^8^, Wen-Qing Li^1^, Wei-Cheng You^1*^, Kai-Feng Pan^1*^**

*** Joint Correspondence to:** Kai-Feng Pan: [pankaifeng2002@yahoo.com](mailto:pankaifeng2002@yahoo.com), Wei-Cheng You:

[weichengyou@yahoo.com](mailto:weichengyou@yahoo.com)

^†^ These authors contributed equally to this work

# Supplementary Tables

Table S1. *Archaea* in two fecal samples

| Sample Number | *H. pylori* status | Gastric lesions | Kingdom | Phylum | Class | Number of sequences | Relative abundances |
| --- | --- | --- | --- | --- | --- | --- | --- |
| 3 | Negative | Normal | *Archaea* | *Thaumarchaeota* | subgroup SCG | 1 | <0.10% |
| 15 | Negative | Metaplasia | *Archaea* | *Thaumarchaeota* | subgroup SCG | 1 | <0.10% |

Table S2. Alpha diversity indices’ distribution of fecal microbiota among different groups

|  |  | Observed species | | | |  | Shannon index | | | |
| --- | --- | --- | --- | --- | --- | --- | --- | --- | --- | --- |
|  |  | Median (IQR) | *p* ^a^ | *p* ^a^ | *p* ^b^ |  | Median (IQR) | *p* ^a^ | *p* ^a^ | *p* ^b^ |
| *H. pylori* infection | |  |  |  | 0.317 |  |  |  |  | 0.696 |
|  | Negative | 317.00  (289.00-328.00) | Reference | - |  |  | 4.74  (3.99-5.27) | Reference | - |  |
|  | Past infection | 351.00  (296.00-362.00) | 0.333 | Reference |  |  | 5.27  (3.46-5.59) | 0.687 | Reference |  |
|  | Current infection | 316.00  (285.75-351.50) | 0.930 | 0.671 |  |  | 4.83  (3.73-5.36) | 0.950 | 0.916 |  |
| Gastric lesions | |  |  |  | 0.377 |  |  |  |  | 0.291 |
|  | Normal | 301.00  (284.00-320.00) | Reference | - |  |  | 4.68  (3.85-4.84) | Reference | - |  |
|  | Gastritis | 327.00  (309.75-351.50) | 0.397 | Reference |  |  | 5.03  (4.26-5.61) | 0.258 | Reference |  |
|  | Metaplasia | 320.00  (281.00-358.00) | 0.759 | 0.910 |  |  | 4.87  (3.42-5.26) | 0.983 | 0.553 |  |
| Activity of gastritis | |  |  |  | 0.114 |  |  |  |  | 0.211 |
|  | Normal | 301.00  (284.00-320.00) | Reference | - |  |  | 4.68  (3.85-4.84) | Reference | - |  |
|  | Non-active | 332.50  (312.25-352.50) | 0.098 | Reference |  |  | 5.03  (4.72-5.61) | 0.184 | Reference |  |
|  | Active | 316.00  (240.00-346.25) | 0.803 | 0.262 |  |  | 4.81  (3.55-5.46) | 0.739 | 0.487 |  |

^a^ Nemenyi test between a specific group and reference group.

^b^ Kruskal wallis test.

Abbreviations: *H. pylori*, *Helicobacter pylori*; IQR, interquartile range.

Table S3. Comparison of fecal microbial community structures among different groups by ANOSIM^a^

| Variables | Compared groups | R | *p* |
| --- | --- | --- | --- |
| *H. pylori* status | Current infection *vs*. Non-current infection | 0.007 | 0.300 |
|  | Past infection *vs*. Negative | 0.316 | 0.004 |
|  | Current infection *vs*. Negative | -0.038 | 0.763 |
|  | Current infection *vs*. Past infection | 0.181 | 0.056 |
| Gastric lesions |  | -0.029 | 0.704 |
|  | Gastritis *vs*. Normal | 0.012 | 0.403 |
|  | Metaplasia *vs*. Normal | -0.041 | 0.600 |
|  | Metaplasia *vs*. Gastritis | -0.037 | 0.904 |
| Activity of gastritis |  | 0.063 | 0.194 |
|  | Non-active gastritis *vs*. Normal | 0.131 | 0.097 |
|  | Active gastritis *vs*. Normal | -0.091 | 0.791 |
|  | Active *vs*. Non-active gastritis | 0.076 | 0.230 |

^a^ ANOSIM, a method based on the Bray-Curtis dissimilarity distance matrices, to test statistically whether there is a significant difference among microbial communities of two or more groups.

Abbreviations: ANOSIM, analysis of similarities; *H. pylori*, *Helicobacter pylori*; *vs*., versus.

# Supplementary Figures


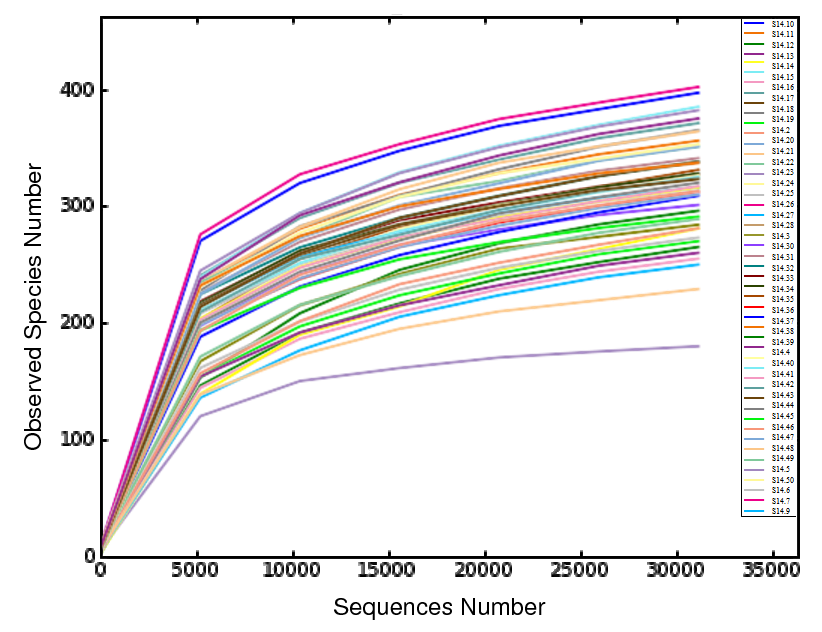


Figure S1. Rarefaction curves of 47 fecal samples.





Figure S2. Associations of fecal microbiota and current or non-current *Helicobacter pylori* infection status. (**A**) Boxplot of observed species in current (n=24) and non-current *H. pylori* infection groups (n=23). (**B**) Boxplot of Shannon index in current and non-current *H. pylori* infection groups. (**C**) Heat-map of the relative abundance distributions of the phyla in current and non-current *H. pylori* infection groups. (**D**) Cladogram of differently distributed taxa between current and non-current *H. pylori* infection groups. The differential taxa with relative abundance less than 0.1% are illustrated between current and non-current *H. pylori* infection groups as ** (*p*<0.01) and * (*p*<0.05), respectively.


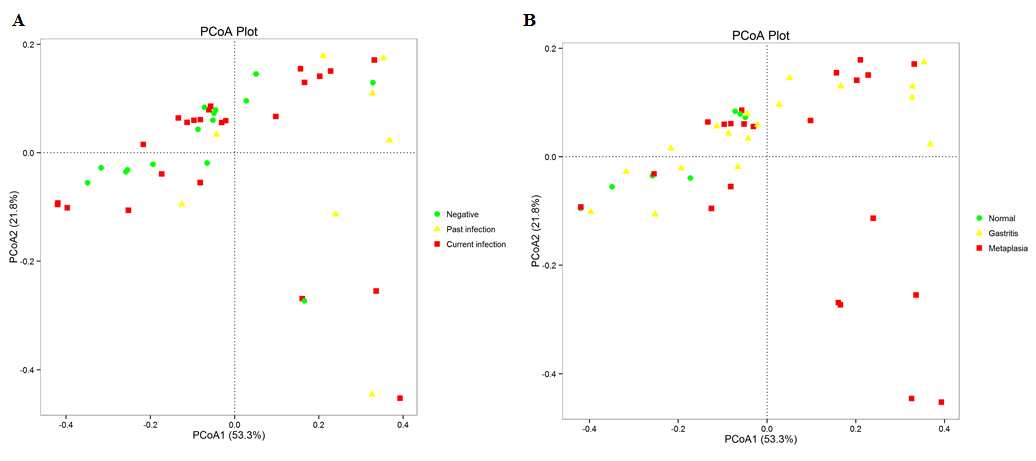


Figure S3. Principal co-ordinates analysis (PCoA) of 47 fecal samples by *H. pylori* status (A) and gastric lesions (B). PCoA was conducted based on weighted unifrac distances of 47 fecal samples. Samples were colored by *H. pylori* status (A) or gastric lesions (B).





Figure S4. Associations of fecal microbiota and activity of gastritis. (**A**) Boxplot of observed species in normal, non-active and active gastritis groups. (**B**) Boxplot of Shannon index in normal, non-active and active gastritis groups. (**C**) Heat-map of the relative abundance distributions of the phyla between non-active gastritis (n=12) and normal (n=7) samples. (**D**) Heat-map of the relative abundance distributions of the phyla in active gastritis (n=6) and normal (n=7) samples. (**E**) Cladogram of differently distributed taxa between active and non-active gastritis groups. The differential taxa are illustrated between active and non-active gastritis groups as * (*p*<0.05).
